# Detection of programmed death ligand 1 protein and CD8^+^ lymphocyte infiltration in plurihormonal pituitary adenomas: A case report and review of the literatures

**DOI:** 10.1097/MD.0000000000009056

**Published:** 2017-12-08

**Authors:** Pengfei Wang, Tingjian Wang, Yakun Yang, Chunjiang Yu, Ning Liu, Changxiang Yan

**Affiliations:** Department of Neurosurgery, Sanbo Brain Hospital, Capital Medical University, Beijing, China.

**Keywords:** growth hormone, immunotherapy, pituitary adenomas, plurihormonal, programmed death protein 1

## Abstract

**Rationale:**

Pituitary adenomas secreting two or more hormones were referred to the plurihormonal tumors. The management of this type of the tumor was tumor resection and amelioration of the unbalanced hormones. However, it washard to cure the plurihormonal adenomas, as they were usually refractory to the traditional treatment. New therapeutic methods were needed in dealing this tumor.

**Patient concerns:**

In this report, we described a patient who suffered from plurihormonal pituitary adenomas, with concomitant prolactin (PRL) and growth hormone (GH) secretion. The adenomas showed aggressive behaviors, which was giant, invasive, and refractory to bromocriptine treatments.

**Interventions:**

The patient underwent a surgical treatment.

**Diagnosis:**

The pathology together with clinical presentation and blood hormone test confirmed that it was PRL-GH secreting tumors.

**Outcomes:**

Although a gross resection was achieved, the postoperative hormones were not normalized. There were also abundant programmed death ligand-1 proteins and CD8^+^ lymphocyte infiltration in the tumor tissues.

**Lessons:**

Our results indicated immunotherapy as a promising treatment for this tumor. More studies were needed to investigate the possibility of the immunotherapy in pituitary adenomas.

## Abstract 2

Supplemental Digital Content is available in the text

## 1 Introduction

Pituitary adenomas accounted for 15.9% of all central nervous system tumors.^[1]^ According to the hormone production, pituitary adenomas were classified into nonfunctioning or functioning adenomas. The functioning adenomas were characterized by the secretion of prolactin (PRL), growth hormone (GH), adrenocorticotropin, gonadotropins, and multiple hormones (plurihormonal).^[2]^ Most of the pituitary adenomas were benign tumor, associated with a favorable prognosis when treated with surgery, medical therapies, and radiotherapy.^[3]^ However, a subset of the pituitary adenomas exhibited aggressive clinical behavior, as they frequently recurred and refractory to the traditional treatment.^[4]^ The aggressive pituitary adenomas were generally invasive, larger, and hormone-secreting (Adrenocorticotropic Hormone, GH, or plurihormonal especially).^[4,5]^ Moreover, it was of great importance to ameliorate the hormone disturbance, which were associated with a higher risk of complications and mortality.^[6,7]^ However, there was limited success in the traditional treatment of aggressive pituitary adenomas, even after the induction of temozolomide.^[8]^

Recently, the immune check point inhibitors, especially anti-PD-1 (programmed cell death protein 1) drugs, had shown great benefits to cancer patients.^[9]^ The predictive biomarkers for PD-1 inhibitors involved the increased expression of the programmed death ligand 1 (PD-L1) proteins and CD8^[+]^ lymphocyte infiltrations.^[10,11]^ Herein, we reported a case of the plurihormonal pituitary adenoma, in which the tumors were detected PD-L1 proteins and CD8^+^ lymphocyte infiltrations.

## 2 Case report

Informed consent was obtained from the patient before the study. All the procedures in our study were approved by the Institutional Review Board of Sanbo Brain Hospital.

**Figure 1:**
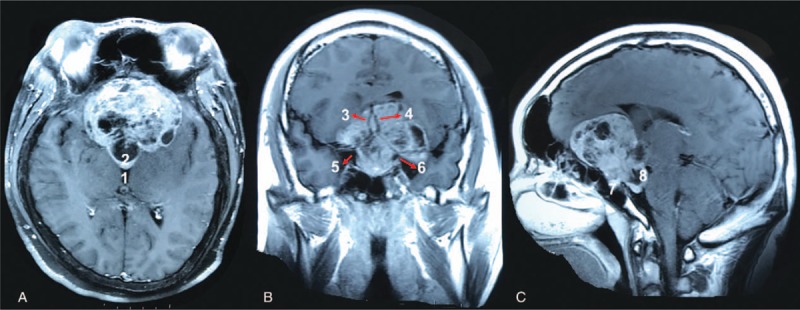
Preoperative magnetic resonance image (MRI) of the pituitary adenomas. The contrast image was presented in axis (A), coronal (B), and sagittal (C) view. 1, third ventricle. 2, The tumors invaded into the third ventricle. 3, A2 segment of the right anterior cerebral artery (ACA). 4, A2 segment of the left ACA. 5, Bifurcation of right internal carotid artery (ICA). 6, Bifurcation of left ICA. 7, Intrasellar tumors. 8, Tumors located at the interpeduncular fossa.

A 27-year-old man complained of a 2-year history of visual loss and right temporal visual field defect, accompanied by headache and nausea. Also he suffered from sex dysfunction for >2 years. Magnetic resonance image (MRI) results and serum PRL levels were consistent with the diagnosis of PRL-secreting adenomas. So he started to take bromocriptine 17.5 mg/day, but in vain. One year later, he stared to present acromegaly and was admitted for operation. Preoperative MRI suggested a giant mass (70 × 52 × 53 mm) in the saddle area (Fig. 1). Radioimmunoassay (Siemens DPC2000) was used for detecting serum GH and PRL. There were significantly increased levels of GH (21.40 >  0–3 ng/mL) and PRL (2901.6 > 2.1–17.7 ng/mL, Supplement Table).

**Figure 2:**
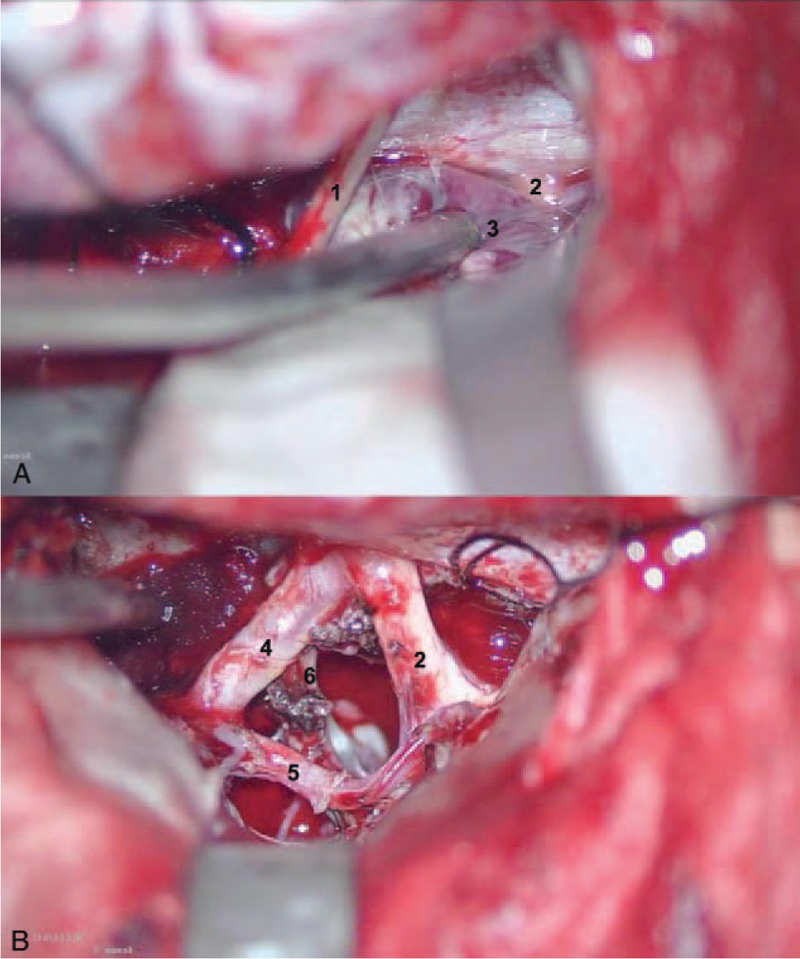
Intraoperative images of left basal interhemispheric approach before (A) and after (B) total resection of the tumor. 1, Olfactory nerve; 2, optic nerve; 3, tumor; 4, internal carotid artery; 5, anterior cerebral artery; 6, oculomotor nerve. All the vessels and nerves were perfectly preserved in the surgery.

The left basal interhemispheric approach was adopted. Intraoperatively, the tumor was tightly surrounded by optic nerves, chiasma opticum, and internal carotid artery and anterior cerebral artery (Fig. 2A). The texture was moderate and the blood supply was rich. The gross removal was achieved with intact surrounding nerves, vessels and brain tissues (Fig. 2B).

Pathological findings proved a pituitary adenoma. The immunohistochemistry was performed as we did previously.^[12]^ Immunostaining of GH and PRL was positive (Fig Supplement 1), suggesting a PRL-GH secreting plurihormonal tumor. Next, the tumor sample obtained from the operation was used to evaluate the PD-L1 expression and CD8^+^ lymphocytes infiltration in the tumor tissues. Excitedly, there were more than 10% tumor cells showing cytoplasmic or membrane's immunostaining of PD-L1 (Cell Signaling Technology, Boston). Also CD8^+^ (BIOCARE) lymphocytes infiltration was observed in the tumor tissues.

**Figure 3:**
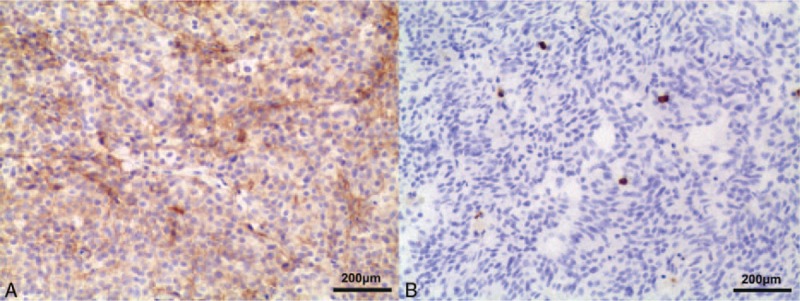
Representative images of pathology. The tumor's cytoplasm and membrane showed immunostaining of programmed death ligand 1 (PD-L1) (A), and the CD8^+^ lymphocytes were infiltrating in the tumor tissues (B).

Postoperatively, the patient had hypopituitarism, diabetes insipidus, and electrolyte disturbance. He was treated with hormone replacement and fluid infusion. The GH (25.20 > 0∼3 ng/mL) and PRL (319.82 > 2.1–17.7 ng/mL, Supplement Table) levels postoperatively were still high. He was discharged 10 days after the operation (Fig. 3).

## 3 Discussion

In this article, we presented a case of plurihormonal pituitary adenoma, in which the PD-L1 proteins and CD8^+^ lymphocytes were detected in the tumor tissues. This tumor showed aggressive behaviors, which was giant, invasive, and refractory to bromocriptine treatments. Postoperative MRI confirmed a gross resection of the tumors. But neither GH nor PRL levels were ameliorated postoperatively. The higher hormones postoperatively were indicative of remaining tumor cells and recurrence.^[13,14]^ So what could we do when tumor recurred? Maybe PD-1 inhibitors could be offered as an alternative opinion in treating this tough tumor.

Tumor microenvironment played a vital role in tumor development. Tumor-infiltrating lymphocytes, macrophages, cytokines, and immune checkpoints were key factors in modulating immune function.^[9,15]^ Recently, there were studies arising concerning the immune microenvironment in pituitary adenomas. There were increased levels of PD-L1 mRNA and proteins in GH or PRL secreting pituitary adenomas, compared with nonfunctioning pituitary adenomas.^[16]^ Both the CD4^+^and CD8^+^ lymphocytes were frequently observed in GH-secreting pituitary adenomas.^[17]^ The inhibition of PD-1 signaling pathways could help CD8^+^ lymphocytes recognize and kill tumor cells.^[15,18]^ Moreover, blockade of the PD-1 signaling pathways was also substantial for macrophages to inhibit tumor development.^[19]^ It was observed a positive correlation of macrophages with tumor's size and invasiveness in pituitary adenomas.^[17]^ So if was reasonable to confer that macrophages could be activated by PD-1 inhibitors, killed pituitary adenomas cells.

The mutation burden and abundant tumor-associated antigen was of great significance in predicting tumor immunotherapy.^[10,11]^ Unluckily, there was a relatively less frequency of genetic mutations in pituitary adenomas, which was not predictive for cancer immunotherapy.^[20]^ However, recent studies suggested radiotherapy and chemotherapy could enhance PD-1 inhibitors’ efficacy in treating tumor.^[21,22]^ Consequently, it might be reasonable to consider that PD-1 inhibitors could be combined with radiotherapy or temozolomide in treating aggressive pituitary adenomas.

## 4 Conclusion

Our reported indicated that immunotherapy as a promising treatment for pituitary adenomas. More studies were needed to investigate the immunosuppressive mechanism in the microenvironment of pituitary adenomas.

## Supplementary Material

Supplemental Digital Content
