# Supplementary material for: Detection of programmed death ligand 1 protein and CD8+ lymphocyte infiltration in plurihormonal pituitary adenomas: A case report and review of the literatures
Source: Medicine (Baltimore). 2017 Dec 8;96(49):e9056. doi: 10.1097/MD.0000000000009056 (PMC5728927; doi:10.1097/MD.0000000000009056)
Supplement: Supplemental Digital Content [file medi-96-e9056-s001.doc]

**Fig. supplement 1** Representative images of GH (a) and PRL (b) immunostaining.


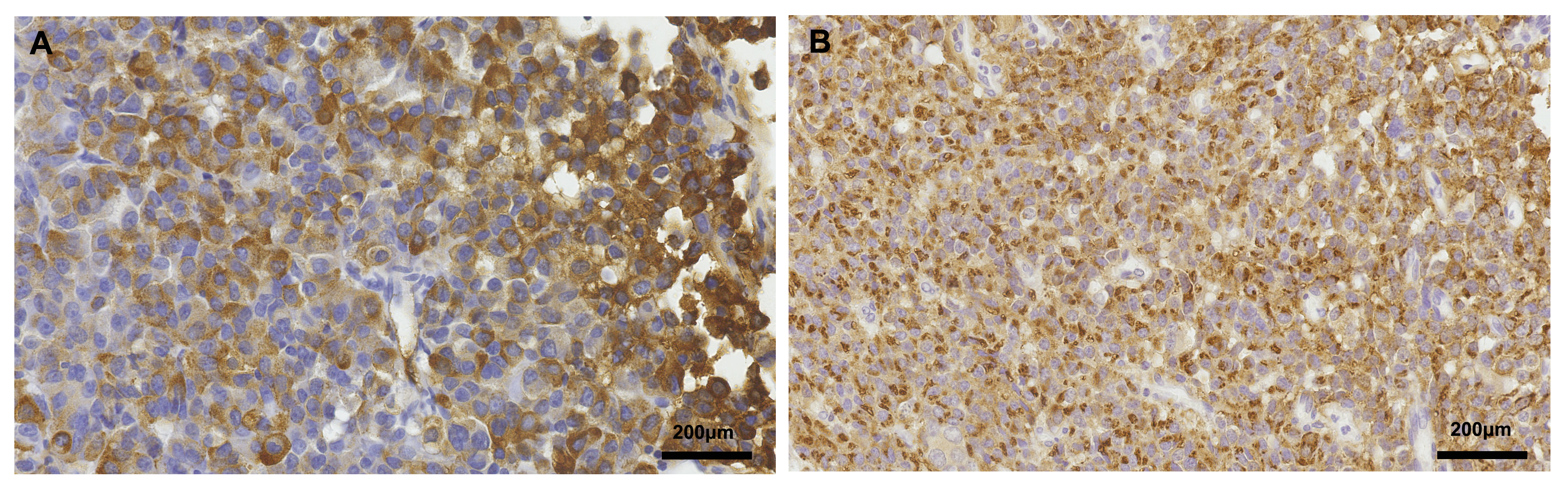


| Supplement table. Perioperative changes in blood hormones of PRL and GH | | | |
| --- | --- | --- | --- |
| Blood hormone | Pre-operation | Post-operation | Normal range |
| PRL | 2901.6 ng/ml | 319.82 ng/ml | 2.1-17.7 ng/mL |
| GH | 21.40 ng/ml | 25.20 ng/ml | 0~3 ng/ml |
